# Who seeks help? Characteristics of doctors accessing mental health support in England: 4-year national review

**DOI:** 10.1192/bjo.2025.10910

**Published:** 2025-12-01

**Authors:** Bhathika Perera, Memta Jagtiani, Louisa Dallmeyer, Ken Courtenay, Sarah Lennard, Rohit Shankar, Angela Hassiotis, Zaid Al-Najjar

**Affiliations:** Division of Psychiatry, University College London, London, UK; NHS Practitioner Health, London, UK; Cornwall Partnership NHS Foundation Trust, Bodmin, UK; Peninsula School of Medicine, University of Plymouth, Plymouth, UK

**Keywords:** Mental health, anxiety, ADHD, doctor, depression

## Abstract

**Background:**

Mental health difficulties affect the well-being of doctors and compromise the delivery of healthcare. However, large-scale data on doctors’ mental health needs are limited.

**Aims:**

Describe patterns of self-referrals for mental health support among doctors in England and explore associations with demographic factors, speciality, neurodevelopmental and mental health indicators.

**Method:**

Observational study using data from doctors who self-referred for mental health difficulties to a national service in England over a 4-year period. Logistic regression was used to explore associations between speciality and mental health indicators.

**Results:**

Of the 16 815 doctors who self-referred during the study period, 80% were under the age of 49 and 70.6% were female with the two largest ethnicities being 65.1% White and 22.7% Asian. Women were more likely to report higher scores for depression (odds ratio 0.90, 95% CI = 0.84 to 0.97), anxiety (odds ratio 0.78, 95% CI = 0.72 to 0.84) and psychological distress (odds ratio 0.78, 95% CI = 0.70 to 0.87), but males were more likely to screen positive for attention-deficit hyperactivity disorder (ADHD) symptoms. Doctors in general practice accounted for 46.3% of referrals. Compared with them, doctors in most other specialities had higher odds of elevated mental health scores across all measures, including ADHD.

**Conclusions:**

The findings highlight a significant mental health burden among self-referring doctors, particularly for females and doctors in non-general practice specialities. Tailored and easily accessible support strategies that account for both demographic and professional contexts are essential to address the diverse mental health needs of the medical workforce.

The mental health and well-being of healthcare professionals is a pressing concern with rising rates of mental disorders and burnout. The World Health Organization, in their 2024 technical consultation, highlighted that at least a quarter of health and care workers reported anxiety, depression and burnout symptoms between January 2020 and April 2022, thus raising concerns about the mental health and well-being of healthcare staff and subsequent threats to the current health systems.^1^ The COVID-19 pandemic, in particular, has spotlighted the importance and impact that providing healthcare can have on staff.^2^ Anxiety, stress, depression and other mental illnesses continue to be the most reported reasons for sickness in National Health Service (NHS) staff accounting for over 25% of all sickness absences in 2024.^3^ Doctors are no exception to the experience of mental health problems despite a career in medicine being considered both rewarding and highly sought after. A systematic review capturing the mental health data of doctors working in North America, Asia, Europe, South America and Africa has shown a high prevalence rate of depression and depressive symptoms up to 43%.^4^ In 2018, a cross-sectional survey of 1651 UK doctors across specialities found that one-third met burnout criteria with those in emergency medicine and general practice suffering the most.^5^ Doctors’ training can be a period of vulnerability with one study demonstrating that 32% of surgical interns develop new-onset depression within the first year of residency.^6^ A survey of 456 resident doctors during the COVID-19 pandemic in the UK found over 40% to be severely depressed, anxious and stressed.^7^ Increased suicide rate, particularly among female doctors (1.05 for male doctors and 1.76 for female doctors), with a decreasing suicide rate with age, has been shown in a systematic review looking at suicide rates among doctors compared with the general population from 20 countries.^8^ Multiple factors, such as lack of control over career advancement, disenfranchisement due to understaffing, negative impacts of shift work and fragmented teams in certain specialities have been reported to increase the risk of mental health difficulties.^9^ Some have argued that increasing mental health problems among healthcare staff are a symptom of the current state of health systems often riddled with staff shortages, low pay, extraordinarily stressful work environments and lack of workplace safeguards.^1^ A study among doctors in the UK found that low job satisfaction, work overload, increased hours worked and neuroticism were associated with mental health problems.^10^ General practitioners (GPs) and consultants reported more symptoms of mental health problems compared with other doctors. Some studies have shown that anaesthetists, psychiatrists and general surgeons are at higher risk of suicide compared with doctors in other specialities.^11^ A study looking at specific neurodevelopmental disorders, such as attention-deficit hyperactivity disorder (ADHD), among surgical resident doctors reported a prevalence rate of 31%.^12^ However, there are no specific studies exploring ADHD in different specialities. A recent study of doctors accessing mental health support found nearly one-third of them screened positive for ADHD.^13^ Mental health difficulties among healthcare professionals extend beyond an individual’s mental and overall well-being. They often impact the delivery of healthcare. Existing evidence shows an association between medical errors, consequent patient safety concerns and the mental health of doctors.^14–16^ Thus, there is a need to establish factors associated with a higher risk of mental ill health among doctors to consider approaches to prevention and treatment.

This study aims to:

- describe the demographic characteristics of doctors experiencing mental health difficulties;
- examine the distribution and factors associated with mental health difficulties and neurodevelopmental conditions across medical specialities and identify which groups may be at higher risk.

## Method

### Study population

In this study, we utilised the NHS Practitioner Health (NHS-PH) database (Appendix 1) between 1 October 2020, and 30 September 2024 (4 years) in England. The database includes data collected by doctors on an online form at the point of self-referral to NHS-PH for mental health support with consent for their data to be included in research. PHQ-9 (Patient Health Questionnaire-9),^17^ GAD-7 (Generalised Anxiety Disorder-7)^18^ and CORE-10 (Clinical Outcomes in Routine Evaluation-10)^19^ were used as screening tools for depression, anxiety and overall psychological well-being. The 6-item Adult ADHD Self-Report Scale v1.1 (ASRS),^20^ a screening tool for ADHD, was added as an indicator of neurodivergence, given a recent study showing a high rate of screening positive for ADHD in this population and its association with anxiety and depressive scores.^13^ Scores of 10 and above for the PHQ-9, 10 and above for the GAD-7, 15 and above for the CORE-10 and 4 or above for ASRS were taken as screened positive.

### Statistical analysis

Descriptive analyses, followed by analytical modelling of the associations between medical speciality and mental health, were conducted. Logistic regression models were used to analyse the odds ratios of scoring high on each mental health indicator for each speciality group compared with general practice, as it was the largest speciality group (the reference group). Odds ratios were analysed for scoring high on PHQ-9, GAD-7, CORE-10 and ASRS for each speciality group compared with the reference group. Gender, age and ethnicity were entered into the models as potential confounders to control for their independent effects on each mental health indicator. The COVID-19 period was used as a variable in the statistical model for all mental health indicators except for ASRS, as ASRS data were only collected post-COVID-19. As some participants contributed repeated measurements, robust standard errors clustered by participant ID was used to account for the non-independence of observations within individuals.

## Results

During the study period, there were 16 815 self-referrals from doctors seeking support for mental health problems (Table 1). Most referrals were from female doctors (70.6%). The largest group seeking support were aged 30–39 (39.6%). The 30–49 age group comprised 69.9% of the referrals. There was a decline in the proportion of doctors seeking support for mental health problems in age 50 and older. The largest ethnic group seeking mental health support was White (65.1%) followed by Asian (22.7%). Thirty-six per cent of the doctors seeking mental health support were recorded as having completed their primary medical qualification (PMQ) in the UK. Those doctors who had obtained their first medical degree from outside the UK comprised 15.4%. However, in nearly 50% of referrals, it was not reported whether they had received a medical degree from the UK or not. The largest group of doctors accessing mental health support were general practitioners (46.3%) followed by general medicine (12.4%) and other specialities (9.8%).

**Table 1:** Characteristics of doctors who registered for mental health support

	Doctors accessing NHS-PH in England, *n* (%)	Doctors in England, *n* (%)
All doctors	16 815 (100.0)	298 800 (100.0)
Gender		
Female	11 872 (70.6)	148 654 (49.8)
Male	4875 (29.0)	150 146 (50.2)
Other	68 (0.4)	
Age		
23–29	695 (4.1)	42 726 (14.3)
30–39	6661 (39.6)	97 862 (32.7)
40–49	5092 (30.3)	71 990 (24.1)
50–59	2520 (15.0)	49 601 (16.6)
60–69	728 (4.3)	26 216 (8.8)
70+	53 (0.3)	10 405 (3.5)
Missing	1066 (6.3)	
Ethnicity		
Asian	3819 (22.7)	102 147 (34.2)
Black	593 (3.5)	22 505 (7.5)
White	10 951 (65.1)	132 322 (44.3)
Mixed	771 (4.6)	8709 (2.9)
Other	486 (2.9)	18 373 (6.1)
Missing/not reported	195 (1.2)	14 744 (4.9)
Primary medical qualification from the UK		
Yes	6048 (36.0)	176 129 (58.9)
No	2596 (15.4)	122 671 (41.1)
Missing	8171 (48.6)	
Speciality		
General practice	7788 (46.3)	
General medicine	2083 (12.4)	
Other	1645 (9.8)	
Psychiatry	966 (5.7)	
Anaesthetics	937 (5.6)	
Surgery	769 (4.6)	
Paediatrics	738 (4.4)	
Emergency medicine	665 (4.0)	
Obstetrics and gynaecology	419 (2.5)	
Radiology	230 (1.4)	
Intensive care	162 (1.0)	
Pathology	127 (0.8)	
Ophthalmology	69 (0.8)	
Public health	55 (0.3)	
Occupational medicine	41 (0.2)	
Unemployed	23 (0.1)	
Missing	98 (0.6)	

NHS-PH, National Health Service Practitioner Health. Specialities were categorised according to the General Medical Council (GMC) main speciality listing. GMC data reflects who is certified as a specialist, while our data reflects the specialities in which doctors are working, regardless of their specialist status; therefore, we did not add the English data to Table 1 for comparison.

The main variables were further analysed using logistic regression to examine their associations with mental health indicators (Table 2). Analysis of different age categories showed that doctors aged 40 and over were less likely to have higher anxiety scores compared with the 30–39 group, with the 40–49 and 50–59 age groups showing statistically significant lower odds (odds ratio 0.87, *p* = 0.001 and odds ratio 0.85, *p* = 0.02), but no statistical significance was observed in the aged 60 and over group. There were higher odds of increased depressive symptoms in the 40–49, 50–59 and 60–69 age groups compared with the 30–39 age group, but they were not statistically significant. The over-70 group had lower odds of depression, but with a wide confidence interval and without statistical significance. The 23–29 age group showed lower odds of increased depressive symptoms (odds ratio 0.88, *p* = 0.246), higher odds of anxiety (odds ratio 10.09 and odds ratio 0.43) and higher odds of increased psychological distress (odds ratio 1.16, *p* = 0.308) compared with the 30–39 age group, which were not statistically significant. All age groups above 40 had lower odds of psychological distress compared with the 30–39 age group. Similarly, all other age groups had lower odds of reporting higher ADHD symptoms compared with the 30–39 age group, except for the 40–49 age group which had an odds ratio of 1.14 but this was not statistically significant (*p* = 0.078). Analysis of gender showed that females were more likely than males to present with higher depression scores (odds ratio 0.90, 95% CI = 0.83–0.98, *p* = 0.012), higher anxiety scores (odds ratio 0.77, 95% CI = 0.72–0.84, *p* < 0.001) and higher psychological distress (odds ratio 0.78, 95% CI: 0.70–0.87, *p* < 0.001) but males were more likely to screen positive for ADHD symptoms (ASRS: odds ratio 1.30, 95% CI = 1.13–1.49, *p* < 0.001).

**Table 2:** Results from logistic regression models for the associations between main variables and four mental health indicators

Characteristics	Model 1 (PHQ)			Model 2 (GAD)			Model 3 (CORE)			Model 4 (ASRS)		
	OR	95% CI	*P*-value	OR	95% CI	*P*-value	OR	95% CI	*P*-value	OR	95% CI	*P*-value
Intercept	1.61	(1.48, 1.75)	<0.001	2.31	(2.13, 2.52)	<0.001	3.31	(2.96, 3.70)	<0.001	0.41	(0.36, 0.47)	<0.001
Gender												
Males (ref. Females)	**0.90**	**(0.83, 0.98)**	**0.012**	**0.77**	**(0.72, 0.84)**	**<0.001**	**0.78**	**(0.70, 0.87)**	**<0.001**	**1.30**	**(1.13, 1.49)**	**<0.001**
Age												
30–39 (ref.)												
23–29	0.88	(0.70, 1.09)	0.246	1.09	(0.88, 1.35)	0.430	1.16	(0.87, 1.54)	0.308	0.90	(0.67, 1.23)	0.518
40–49	1.06	(0.97, 1.15)	0.188	**0.87**	**(0.80, 0.95)**	**0.001**	0.90	(0.80, 1.01)	0.074	1.14	(0.99, 1.33)	0.078
50–59	1.10	(0.99, 1.23)	0.072	**0.85**	**(0.76, 0.94)**	**0.002**	0.94	(0.81, 1.10)	0.454	0.87	(0.72, 1.06)	0.171
60–69	1.09	(0.91, 1.30)	0.350	0.86	(0.73, 1.02)	0.089	0.92	(0.71, 1.20)	0.559	0.87	(0.61, 1.23)	0.426
70+	0.87	(0.49, 1.55)	0.641	0.78	(0.44, 1.38)	0.391	0.63	(0.29, 1.39)	0.255	0.51	(0.11, 2.46)	0.403
*P*-value			0.450			**0.002**			0.336			0.078
Ethnicity												
White (ref.)												
Asian	**1.10**	**(1.01, 1.20)**	**0.029**	1.04	(0.95, 1.13)	0.413	**0.87**	**(0.78, 0.98)**	**0.025**	1.06	(0.91, 1.23)	0.456
Black	0.99	(0.82, 1.21)	0.948	**0.66**	**(0.54, 0.79)**	**<0.001**	**0.78**	**(0.60, 1.00)**	**0.050**	0.96	(0.69, 1.33)	0.792
Mixed	1.16	(0.97, 1.39)	0.097	1.01	(0.85, 1.20)	0.911	1.21	(0.94, 1.56)	0.144	1.17	(0.86, 1.59)	0.307
Other	**1.74**	**(1.38, 2.19)**	**<0.001**	**1.66**	**(1.32, 2.08)**	**<0.001**	1.30	(0.97, 1.75)	0.083	**2.15**	**(1.56, 2.96)**	**<0.001**
*P*-value			**<0.001**			**<0.001**			**0.003**			**<0.001**
COVID-19 period												
Yes (ref. No)	1.00	(0.93, 1.07)	0.987	0.98	(0.92, 1.05)	0.592	0.93	(0.82, 1.05)	0.238	–	–	–
Speciality												
General practice (ref.)												
Anaesthetics	**1.54**	**(1.31, 1.81)**	**<0.001**	1.13	(0.96, 1.32)	0.130	1.18	(0.95, 1.46)	0.141	1.30	(0.98, 1.71)	0.068
Emergency medicine	**1.71**	**(1.41, 2.07)**	**<0.001**	1.17	(0.97, 1.41)	0.095	**1.83**	**(1.37, 2.44)**	**<0.001**	**1.79**	**(1.33, 2.43)**	**<0.001**
Intensive care	**1.95**	**(1.33, 2.86)**	**<0.001**	1.18	(0.83, 1.68)	0.343	**2.66**	**(1.47, 4.80)**	**0.001**	1.18	(0.67, 2.06)	0.569
General medicine	**1.53**	**(1.36, 1.71)**	**<0.001**	**1.16**	**(1.04, 1.30)**	**0.010**	**1.44**	**(1.22, 1.69)**	**<0.001**	1.05	(0.86, 1.28)	0.627
Obstetrics and gynaecology	**1.80**	**(1.41, 2.29)**	**<0.001**	1.05	(0.84, 1.31)	0.691	1.09	(0.80, 1.49)	0.569	1.16	(0.79, 1.71)	0.455
Occupational medicine	1.15	(0.58, 2.26)	0.692	1.15	(0.57, 2.32)	0.691	1.23	(0.49, 3.07)	0.658	**5.98**	**(1.62, 22.12)**	**0.007**
Ophthalmology	1.22	(0.71, 2.10)	0.477	1.25	(0.69, 2.27)	0.459	1.27	(0.53, 3.01)	0.593	1.00	(0.30, 3.35)	0.998
Paediatrics	**1.41**	**(1.18, 1.68)**	**<0.001**	1.15	(0.97, 1.37)	0.111	1.21	(0.95, 1.52)	0.120	**1.41**	**(1.04, 1.90)**	**0.028**
Pathology	**1.98**	**(1.21, 3.23)**	**0.006**	0.90	(0.60, 1.35)	0.595	1.02	(0.60, 1.73)	0.937	1.50	(0.74, 3.05)	0.262
Psychiatry	**1.46**	**(1.25, 1.70)**	**<0.001**	1.10	(0.94, 1.28)	0.248	**1.43**	**(1.14, 1.80)**	**0.002**	**1.49**	**(1.14, 1.94)**	**0.003**
Public health	**2.49**	**(1.24, 5.01)**	**0.011**	**2.07**	**(1.04, 4.10)**	**0.038**	2.07	(0.81, 5.30)	0.128	1.21	(0.36, 4.05)	0.757
Radiology	**1.40**	**(1.02, 1.92)**	**0.040**	1.12	(0.82, 1.52)	0.491	**1.62**	**(1.02, 2.60)**	**0.043**	1.27	(0.75, 2.13)	0.373
Surgery	**1.84**	**(1.52, 2.21)**	**<0.001**	**1.21**	**(1.02, 1.44)**	**0.029**	**1.42**	**(1.11, 1.82)**	**0.005**	1.10	(0.82, 1.49)	0.510
Other	**1.71**	**(1.46, 2.00)**	**<0.001**	1.02	(0.88, 1.19)	0.758	1.19	(0.95, 1.50)	0.130	**1.79**	**(1.36, 2.37)**	**<0.001**
*P*-value			**<0.001**			0.102			**<0.001**			**<0.001**

PHQ, Patient Health Questionnaire; GAD, generalised anxiety disorder; CORE, Clinical Outcomes in Routine Evaluation; ASRS, Adult attention-deficit hyperactivity disorder Self-Report Scale; OR, odds ratio; ref, reference category. Model 1: Observations: *n* = 15 383. Clusters (participants): *n* = 13 620. Model 2: Observations: *n* = 15 371. Clusters (participants): *n* = 13 609. Model 3: Observations: *n* = 9187. Clusters (participants): *n* = 8623. Model 4: Observations: *n* = 4554. Clusters (participants): *n* = 4434.

Bold data indicate statistically significant differences (*p* < 0.05) compared with the reference group.

Compared with the reference group of general practice, all other specialities had higher depression scores that were statistically significant except in occupational medicine (odds ratio 1.15, *p* = 0.692) and ophthalmology (odds ratio 1.22, *p* = 0.477). Anxiety scores were higher with statistical significance among doctors in general medicine (odds ratio 1.16, *p* = 0.010), public health (odds ratio 2.07, *p* = 0.038) and surgery (odds ratio 1.21, *p* = 0.029) compared with general practice. Doctors in emergency medicine, intensive care, general medicine, psychiatry, radiology and surgery reported higher levels of psychological distress, with statistical significance, compared with those in general practice. Doctors in emergency medicine, occupational medicine, paediatrics, psychiatry and other specialities had higher odds of screening positive for ADHD with statistical significance compared with general practice.

## Discussion

Understanding mental health problems among doctors is essential to provide effective treatment and support as well as appropriate interventions that address systemic factors contributing to these issues. This study provides important insights into the demographic, professional characteristics and indicators of mental health and neurodevelopmental conditions of doctors seeking support for mental health problems. To discuss these associations, it is important to compare the results with data for all doctors in England.^21^

Our study shows that a large number of referrals from doctors (43.4%) were from those under the age of 40 years. This falls within the first 10–15 years of a medical doctor’s career, a stage marked by increased demands and challenges. However, General Medical Council (GMC) data confirms that 47% of doctors practising in England are under the age of 40,^21^ which suggests that the number of doctors seeking support is proportionate to the number of doctors in those age categories. In England, 50.2% of the doctors were male and 49.8% were female. However, in our study, a higher percentage of referrals (70.6%) were from females. This may align with previous research suggesting that women are more likely to seek help for mental health concerns^22^ or that women are at a higher risk of mental health conditions. Previous research also indicates that women are more likely to experience common mental disorders compared with men^23^ but the higher percentage in our study could also be due to gender-based diagnostic biases. In addition, our study demonstrates that female doctors were also more likely to present with lower mood, higher levels of anxiety and increased psychological distress scores compared with male doctors. Male doctors were more likely to screen positive for ADHD compared with female doctors. Even though ASRS is not diagnostic of ADHD, the increased odds of screening positive for ADHD among male doctors highlights the need for improved awareness of neurodivergence among doctors.

There is debate about whether doctors who complete their PMQ outside the UK account for differential attainment in their medical careers.^24^ GMC data indicates that 41.1% of the current medical workforce gained their PMQ outside the UK. In our study, among the doctors who provided the data, 15.4% reported that their PMQ was outside of the UK medical schools. A possible reason for the lower referral rate from doctors with PMQs from non-UK medical schools may be a lack of awareness of this service, changes in the trends in the number of doctors with non-UK PMQs coming to the UK over the years, particularly the increasing numbers in younger age groups or owing to differences in help-seeking behaviour. A recent study based on GMC data showed a lower risk of work-related burnout among doctors with non-UK PMQs compared with those with UK PMQs, which may also help explain the lower referral rate in this group.^25^

The largest percentage of doctors (65.1%) were of White ethnicity in the study. However, 44.3% of the medical workforce in England is of White ethnicity.^21^ The percentage of doctors of Asian or Black ethnicity referring themselves to NHS-PH (Asian: 22.7%, Black: 3.5%) was lower than the percentage of Asian and Black doctors working in England (Asian: 34.2%, Black: 7.5%). This may raise questions about potential cultural or systemic barriers to accessing mental health support among doctors from minority groups. Ethnic minority groups face reduced access to confidential or culturally competent mental health services.^26^ Stigma around mental health in some minority communities may discourage help-seeking, particularly in a professional context where vulnerability could be perceived as weakness.^27^

The majority of the referrals were from general practice (46.3%). Whether doctors in general practice are more likely to experience mental health problems given the high workload, emotional burden and isolation often reported in primary care or more likely to seek support for mental health problems, are possible explanations. The NHS-PH has been rooted in primary care since its inception and first expanded to offer access to GPs in England before expanding to doctors in secondary care. As the NHS-PH has been available to GPs for a longer period, it is possible that, as a group, they have a greater awareness of the service compared with other specialists so that help-seeking behaviours may have become more normalised within this population. This cultural shift has enabled earlier intervention, reducing the risk of issues escalating to a crisis point. In contrast, those working in secondary care, who have only more recently gained access across England, may still experience greater barriers to seeking support. The lower rate of referrals from doctors working in specialities such as psychiatry (6%), surgery (5%) and paediatrics (4%) needs further exploration as it may not be a reflection of a lower rate of mental health difficulties in these groups considering US studies where up to 78% of psychiatrists experienced burnout and 16% scored high on PHQ-9 for depression.^28^ We have not been able to compare the percentages of doctors in different specialities to the GMC database, as the GMC speciality data is based on the number of doctors holding specialist registration rather than those working in them.

The higher odds of increased depression scores for doctors in many specialities achieving statistical significance (anaesthesia, emergency medicine, intensive care, general medicine, obstetrics and gynaecology, paediatrics, pathology, psychiatry, public health, radiology, surgery and others) compared with doctors in general practice may be due to many factors. They include whether doctors in hospital specialities are less likely than doctors in general practice to seek mental health support early on when experiencing mental health difficulties. It could be argued that a significant proportion of the workload of doctors in general practices involves consulting with patients with mental health problems. Therefore, they may be more attuned to recognising early warning signs in themselves and more likely to seek support at an earlier stage. The current evidence base does not explain why the highest odds ratio for depression and anxiety was recorded among doctors in public health (odds ratio 2.49 and 2.07, respectively). Possible reasons may relate to work-related factors, such as leadership responsibilities during the COVID-19 pandemic and moral distress, as well as systemic factors, including structural changes in the English public health system. We are cautious in discussing possible reasons why doctors working in emergency medicine, intensive care, general medicine, psychiatry, radiology and surgery are more likely to report higher levels of psychological distress compared with doctors in general practice. However, potential factors to consider are frequent exposure to trauma, critical illness, death, limited opportunities to establish longer-term relationships with patients like in general practice, chronic staffing shortages, the nature of shift work and a culture of reluctance to seek help.

High odds for screening positive for ADHD among doctors in emergency medicine, occupational medicine, psychiatry, paediatrics and other specialities is an unexpected finding, but needs to be explored in future studies to assess whether a pattern is emerging that may suggest decisions to follow different specialities are shaped by neurodevelopmental conditions. Furthermore, better recognition of ADHD-like symptoms in this group of patients, along with other mental health conditions that can mimic ADHD is needed. Caution is needed when interpreting these odds ratios, as they can overestimate risks when a condition has a higher prevalence. This is particularly important with screening for ADHD, as previous studies have shown that nearly one-third of doctors with mental health problems screen positive for ASRS.^13^

It is worth noting that the study period overlaps with the COVID-19 pandemic, which had a profound impact on healthcare delivery. However, no statistically significant difference was observed when the COVID period was included as a variable in the statistical modelling. This may be attributable to a latency effect of COVID-19 related mental health problems as the impact on mental health may have emerged later. Further longitudinal follow-up studies may help to explore these potential delayed effects.

### Strengths and limitations

This study represents the largest analysis to-date of doctors accessing a national mental health support service in England. The findings offer valuable insights and suggest several avenues for future research aimed at deepening the understanding of mental health challenges among medical professionals, particularly through the use of large-scale data-sets. While the size of the data-set is a notable strength, the use of routinely collected administrative data introduces certain limitations. The reliance on self-referral data may introduce selection bias, as those who seek help may differ systematically from those who do not. For example, individuals who are more comfortable seeking help may be over-represented in the data-set.

The high proportion of missing data on PMQs also limits the ability to draw firm conclusions about international medical graduates. A sensitivity analysis using multiple imputation was not performed because of the likelihood that the data were not missing at random (e.g. due to discomfort with the question), so it may have implications for the generalisability of our findings. Additionally, the cross-sectional nature of the data precludes causal inferences. The mental health screening tools employed in this study also have inherent limitations in their sensitivity and specificity to detect mental disorders. Furthermore, despite the confidential nature of the service, some doctors may have been reluctant to disclose sensitive information, which could have potentially affected the completeness of various data fields. Finally, as the study is limited to individuals who presented to NHS-PH, the findings cannot be generalised to the broader population of doctors, nor can they be used to estimate the true prevalence of mental health issues or associated risk factors within the profession. Other important variables, such as substance misuse and shift-work patterns, were not explored in this study. Future research should investigate these factors further to understand the underlying drivers of mental health problems.

### Implications for policy and practice

These findings have several implications for medical workforce policy and support systems:

Medical Education: the higher prevalence of mental health difficulties among younger doctors suggests that early career support programmes need to be enhanced, particularly during the transition from medical school to clinical practice.

Workplace Interventions: the speciality-specific patterns identified suggest that mental health support should be tailored to the unique stressors of different medical fields. Certain specialities showing elevated psychological distress may benefit from focused studies to understand the reasons for these findings compared with other specialities.

Gender-Sensitive Approaches: the differential presentation of mental health symptoms by gender indicates a need for targeted support and intervention strategies.

Diversity and Inclusion: the underrepresentation of Asian and Black doctors in help-seeking behaviour may suggest the need to consider culturally sensitive outreach strategies, which may include peer support programmes and multilingual resources.

Service Delivery: the finding that hospital doctors present with more severe symptoms than doctors in general practice possibly suggests delayed help-seeking in secondary care. This indicates a need for proactive mental health support within hospital settings as a preventative measure. Workforce well-being policies should take these factors into account to better support the mental health of hospital-based staff.

### Summary

There were differences in gender, ethnicity and medical specialities among doctors who sought support for mental health difficulties. The overrepresentation of female doctors and doctors in general practice, alongside the underrepresentation of certain ethnic groups, particularly Asian and Black doctors, raises important questions regarding help-seeking behaviours and the potential disparities in mental health risk across different groups within the medical profession.

Most hospital doctors scoring high on mental health indicators at the point of seeking help, compared with doctors in general practice may reflect earlier help-seeking behaviours in the latter group, linked to longer-standing access to dedicated support. In contrast, hospital doctors, only more recently eligible for similar services, may still face cultural and structural barriers, contributing to delayed and therefore higher indicators at the point of entry. Similarly, further exploration is needed to understand why more male doctors compared with female doctors and some hospital-based speciality doctors compared with doctors in general practice screened positive for ADHD. These findings also highlight the importance of considering the heterogeneity of the medical workforce when delivering mental health services. Tailored, culturally sensitive and accessible support mechanisms are essential to improve early engagement, particularly among groups less likely to seek help proactively and who may present with more severe mental health conditions at the point of accessing care.

## Data Availability

The data supporting the findings of this study are available upon reasonable request by submitting an application to National Health Service Practitioner Health (NHS-PH) directly.

## Appendix 1

NHS Practitioner Health is a unique and confidential service for doctors and dentists in London, UK. Since its inception in 2008, it has grown over the years, providing services to all NHS staff members in England who experience barriers to mental healthcare through local services. This specialist service for the growing mental health needs of doctors has been welcomed, especially with increasing recognition that doctors experience significant barriers to accessing mental health care because of self and perceived stigma, lack of awareness, confidentiality concerns and lack of time to seek help.^27,28^ As the only dedicated service for doctors with mental health problems in England, NHS-PH holds data that can provide insight into the current mental health of doctors in the country.
