# Who seeks help? Characteristics of doctors accessing mental health support in England: 4-year national review – CORRIGENDUM

**DOI:** 10.1192/bjo.2026.12050

**Published:** 2026-06-26

**Authors:** Bhathika Perera, Memta Jagtiani, Louisa Dallmeyer, Ken Courtenay, Sarah Lennard, Rohit Shankar, Angela Hassiotis, Zaid Al-Najjar

This article was originally published with a typographical error in the Methodology section.

The sentence ‘In this study, we utilised the NHS Practitioner Health (NHS-PH) database (Appendix 1) between 1 October 2020, and 30 September 2024 (4 years) in England.’ is incorrect.

The sentence should read:

In this study, we utilised the NHS Practitioner Health (NHS-PH) database (Appendix 1) between 1 October 2019, and 30 September 2023 (4 years) in England.

The authors apologise for the error.
